# Path of excellence: A co-authorship network analysis of European Research Council grant winners in social sciences

**DOI:** 10.1016/j.heliyon.2024.e32403

**Published:** 2024-06-19

**Authors:** Anna Urbanovics, István Márkusz, Gergely Palla, Péter Pollner, Péter Sasvári

**Affiliations:** a Faculty of Public Governance and International Studies, Ludovika University of Public Service, Ludovika Square 2, Budapest, H-1083, Hungary; b Dept. of Biological Physics, Eötvös Lóránd University, Pázmány P. stny. 1/A, Budapest, H-1117, Hungary; c MTA-ELTE Statistical and Biological Physics Research Group, Pázmány P. stny. 1/A, Budapest, H-1117, Hungary; d Health Services Management Training Centre, Semmelweis University, Kútvölgyi út 2, Budapest, H-1125, Hungary; e Faculty of Mechanical Engineering and Informatics, University of Miskolc, Miskolc-Egyetemváros, H-3515, Hungary

**Keywords:** European research council, Social sciences, Co-authorship network, Community finding, Research excellence

## Abstract

The international scientific community puts an ever-growing emphasis on research excellence and performance evaluation. So does the European Union with its flagship research excellence grant scheme organised by the European Research Council. This paper aims to provide an in-depth analysis of one of the ERC's thematic panels within the social sciences, namely the SH2 “Political Science” panel. The analysis is based on empirical, statistical methods, and network analysis tools to gain insights about the grant winners' publication patterns and their coauthor networks. The results draw up an academic career track of the grantees based on quantitative publication patterns and performance. Besides, a change in authorship can be observed, which is proven by the formation of new groups and intensifying intra-group collaboration patterns in the case of all three grant types. However, the ERC grant serves different functions for the winners of three different categories: for the Starting Grant winners, it offers the possibility to kick off and establish their research group, for the Consolidator Grant winners, it opens up new opportunities to extend their co-authorship network, and for the Advanced Grant winners, it offers the chance to start a new collaboration.

## 1 Introduction

The features and priorities of the international academic community are influenced by various international processes. The newest and most significant difference in scientific policies in transition is that they are increasingly based on scientific excellence. So far, scientific excellence has not been universally defined; each country defines it in terms of its individual values and interests. This research focuses on international grants for research excellence, based on the case study of the European Union's flagship grant scheme, the European Research Council (ERC).

Whether the research excellence and in alignment with this, the grant's success can be measured solely by bibliometric indicators is still questionable, because there are contradictory data on their value as a measure of success [1,2]. Besides these, the value of bibliometric data varies between research disciplines as well [3]. However, studies indicate a high correlation between grant success and research performance measured by bibliometric data [4,5]. One of the studies proposed the analysis of co-authorship networks as an alternative solution to measure grant success [6], because it does not solely measure individual research performance but puts it in a wider social context [7,8]. That paper analysed a cohort of junior and senior life sciences' ERC grantees from the years 2007–2009, both the publications/citations outputs and the collaboration networks in the 5-year period before and after the award of their grants. The findings pointed out the added value of the network analysis joined to the bibliometric analysis in the case of grant winners. The authors found altered benefits of ERC grants for junior and senior researchers. For junior researchers, they observed an increase in the number of publications after the ERC grant, especially in the number of last author publications, and the expansion of their collaboration networks. For senior researchers, the authors found that the centrality of their collaborative networks decreased after the grant. This can be translated as a positive effect due to their growing collaboration network, where they play the less significant role of the last author, so they are included in more projects, where they do not necessarily contribute to a great extent. The effect of co-authorship networks is widely studied. In general, we can see a positive correlation between productivity and the number of co-authors, suggesting that with more publications, opportunities arise for having more co-authors involved and for more papers written in common with each co-author [9,10]. This relationship follows a power law. Similar results have been found not only at the individual level [11,12], but also for institutions [13,14].

Experts consider the ERC's performance and achievements to be a success. Grants provide an opportunity for early-stage researchers to break out of the constraints of academic hierarchy and become independent enough to start creating and networking on their own [15]. On the other hand, the system allows more experienced professors to receive funding for their research, which might be riskier but promise real scientific breakthroughs at the same time. Grant winners also emphasise a major change in their visibility and prestige after winning the ERC grant both externally and within their host institution [16].

This research aims to quantify what the excellence defined by the ERC grant means and map out the career path leading to it, in the case of the Social Sciences and Humanities group 2 (SH2 group). This group consists of social scientists in the field of law, communication, sociology, social anthropology, political sciences, and human-technology relations. We also aim to provide an overview of the changes in the co-authorship network around Grantees after the award. Therefore, we analysed a cohort of ERC grantees between 2007 and 2018 winning Starting, Consolidator, or Advanced ERC Grant in the SH2 group. For the analysis, we built up a database containing their bibliometric data, including publication and citation outputs exported from the Scopus database. For the network, we collected the same data of their co-authors based on their common publications. The final database contains more than 14 thousand authors’ Scopus records and bibliometric data.

In alignment with these research aims the research questions are the following.

1. Can we identify leading countries and institutions based on the number of researchers who have won ERC grants in the sample studied?
2. What are the main publication patterns of ERC grant winners in SH2? Can we identify a career path of successful applicants?
3. How does the grant affect the publication patterns and the co-authorship network around the winner?

After the introduction, our study continues with the description of the context of the research, including the concept of research excellence, forms of research excellence policies, the European Research Area, and forms of ERC grants. After these, in Section 3, we describe the methodology used and data sources. In Section 4, the research results are presented, then in Section 5, we discuss these findings in alignment with the literature. Finally, we draw conclusions while taking into account the limitations of empirical analysis.

## 2 Context of the research: the research excellence

Research excellence is yet to be universally defined. Among several alternatives, the following definition may best fit the topic and objectives of this study [17]:

“Research excellence is the ability of a researcher or institution to have a significant impact on a given discipline by leading other researchers to ask new questions and contribute new, significant, and useful results to the body of common existing knowledge using new methods. Research excellence should be demonstrated through a variety of means (e.g., publications, citations, research grants) and should be recognised by the scientific community through honours, prizes, or other rewards.”

There are two main concepts about the constituent elements of research excellence. On the one hand, some authors think that research impact and research quality are the same elements [18], while others distinguish between these two elements [19].

At the EU level, the concept of excellence has gone through significant changes and become sharply defined as being related to breakthrough research, which policymakers attempt to measure and promote with quantifiable indicators [20].

A Slovenian study aimed to measure the concept of scientific productivity and scientific excellence within a small scientific community based on the example of Slovenian researchers. They found that collaboration, mainly collaboration beyond borders, has a strong positive effect on both productivity and excellence, while these aspects and underlying mechanisms cannot be measured based solely on bibliometric indicators. Besides these, their results coming from interviews with Slovenian researchers pointed out significant differences between the attitude of researchers in different disciplines towards excellence. Mainly the researchers in humanities, arts, and social sciences emphasised that research excellence and quality are far beyond the scope of quantitative indicators [21].

### 2.1 Forms of research excellence policy

Most countries have some policies and programmes for research excellence. The ERC, with its project-based and competition-enhancing nature, does not differ from these country-level initiatives. The uniqueness of the ERC is that it implements a research excellence strategy at the European research level [22]. It is apparent that in the dynamically transforming international scene, innovation has become one of the most important domains both in basic and applied research. Research excellence can be motivated by various tools at both national and international levels, and each state can establish these based on their respective priorities and resources.

Demeter investigates academic productivity and academic capital, stating that peer-reviewed articles have become the most significant “*currency of business*” [23]. The concept of academic capital originated from Bourdieu's book titled “*Homo Academicus*” [24]. In this book, he provides analysis on the academic sphere but describes it not only as a forum for dialogue but also as a power sphere where individual career paths are built, challenged, or even destroyed. The academic capital comes not only from a restricted number of pillars but also from a wide range of features contributing to a very complex phenomenon. These factors are the social origins, current position, academic relations, the number of articles published and where they are published, political engagements, and media presentations. The concept allows us to see that academic capital depends not only on one's skills and research productivity, but also on the general reputation factors. Related to the concept of academic capital, we can quote here the five laws of Albert-László Barabási. In his book titled “*The Formula: The five laws behind why people succeed*”, he lists the five most important factors in a form of general laws contributing to academic success [25]. These laws are tested by network science tools, providing good examples for the success of the sport, academic, and artistic career paths.

The five laws are as follows.

1. Performance drives success, but when performance cannot be measured, our networks drive success.
2. Performance is bounded, but success is unbounded.
3. Future success is dictated by the previous success multiplied by the fitness of your idea or product.
4. While team success requires diversity and balance, a single individual will receive credit for the group's achievements.
5. With persistence, success can come at any time.

However, as it has been introduced in the theoretical background section, the university rankings place a strong emphasis on the research productivity of institutions and academics. These articles dominate and influence the international university rankings more than anything else. With these performance rankings, not only higher education institutions, but also entire national higher education systems become measurable, comparable, and transparent. Sidorenko and Gorbatova begin their study with the statement that these rankings not only measure success but also introduce a huge challenge to higher education players and nations in the pursuit of a better rank [26].

A major form of evaluating research excellence is the grant-based system. An example of such a system is the ERC grant scheme established by the European Union. The European Research Council is unique in the aspect that it offers a scheme funded by the European Union for the best researchers from the Member States, alongside strategies at the European nation-state level. This creates a common area of research in Europe on the one hand, while driving Member States to cooperate and compete with one another, increasing their competitiveness. The requirements for research excellence are not unique for the ERC, but they can also be found in other countries as well, for example, in the United States (NIH and NSF).

### 2.2 Common European research area

The definition for the European Research Area (ERA) was set up by the EU in 2000, in parallel with the Lisbon Treaty/Lisbon Strategy, as one of its most prominent objectives. The Strategy aims to create the most competitive and dynamic knowledge-based economy possible. Since then, the scope of the ERA has been expanding and its key subjects increasing, which now include certain environmental and social issues as well. As of now, the ERA is increasingly promoting excellence in national scientific organisations which is reflected in more efficient knowledge sharing and transfer on the one hand, and in the management of social problems on the other.

The priorities of the ERA are the following.

- more efficient national research institutions;
- optimal cooperation and competition at a transnational level that includes research infrastructure;
- free labour market for researchers;
- gender equality in the academic and scientific labour market;
- ensuring optimal access to knowledge, i.e., “open access”;
- international cooperation.

The ERC is the first umbrella organisation that operates at a supranational level, its main focus being the formation of a homogeneous European research elite consisting of the best scientists, rather than international relations and networking [27]. The key point in this approach is that the organisation does not seek to implement all of the national research and development strategies, but rather to create its own, European level of strategy, which is based on international cooperation, representing the interests and values of the European Union [28].

The ERC can be the solution to one of the major challenges of European countries and turn one-way brain drain (especially the migration of European scholars and researchers to the United States) into a retroactive process [29]. Regarding this, the main objectives are defined as follows.

- Educate, attract, and discourage the migration of outstanding talents;
- Consolidate the efforts of national strategies into a common EU strategy;
- Inspire and motivate young researchers;
- Develop attractive career paths;
- Create and establish the standards for a European “*Champion's League*” for research.

The ERC is the only European research organisation or initiative that focuses solely on the measure of research excellence. The ERC creates a certain European value addition that comes from EU-level competition. Along with the activities of the ERC, a new European research elite will emerge from its grant winners. Research excellence cannot be given a general definition, but it becomes truly meaningful and valid when it is recognised. The ERC has a strong normative impact, which means that it can influence international organisations as well [30].

According to Thomas König [31] “*European Research Council* has become the most revered instrument in European science policy and one of the world's most important focal points for the funding of scientific research”. Based on the available statistics, 20 % of the projects supported by the ERC led to a scientific breakthrough, 79 % of them achieved major scientific results, while more than 50 % of these projects had a major economic and societal impact [32]. A distinguishing feature of the ERC is that the panel members and reviewers are indeed international, meaning that they come from various countries, providing the highest quality in the review process, and in return, they can read first-hand the latest developments in their respective fields. The ERC also cultivates an academic culture based on multiculturality [33,34]. Due to its impact and achievements, the ERC has truly become a benchmark quality indicator at the European level.

## 3 Methods

In this paper, we analysed the ERC grant scheme of the SH2 group (Social Sciences and Humanities – Development, economic growth). The list of grant winners was obtained from the official website of the ERC Funded Projects, including the name of the grant winner (principal investigator), the affiliation, the grant type, and the period of the grant. Data collection was restricted to the three main grant types, namely the Starting-, the Consolidator-, and the Advanced Grant. Scopus IDs were determined for each winner using their names, their affiliations (at the time of grant application), and the discipline of corresponding publications. The publication profiles – including the number of papers, the number of citations, and the value of H-index – were collected from the Scopus (international multi-disciplinary citation database of Elsevier) based on the Scopus author IDs. Throughout the text, the nationality of the winners refers to that of their host institution. The lists of publications were also exported from the Scopus database to further analyse the publication patterns, with respect to the authorship roles and collaboration systems. The analysed sample consists of 353 researchers in the SH2 group, including every successful grant winner in the programme since its launch in 2008.

Data analysis was carried out using Excel and Python (using networkx and standard data science packages such as pandas, numpy, and statsmodels), while the figures were made with Cytoscape, Excel and the matplotlib Python package. Before calculating averages, the data was cleaned by discarding points further from the median than the interquartile range (IQR). This method was carried out separately for each analysis.

To examine the research questions from multiple aspects, we also analysed the co-authorship network around the ERC winners, following the concepts of network science, corresponding to a multidisciplinary approach for the study of complex interconnected systems [[35], [36], [37]]. For this analysis, in addition to the publications of the 353 ERC winners mentioned above, all the papers of their co-authors were collected as well. The co-authorship network was built via taking the authors as nodes and drawing a link between pairs who published a paper together. For any given co-author pair, each publication contributed to the weight of the link with 1/(N −1), where N corresponded to the number of authors in the paper. Thus, papers with many authors did not contribute as much to the strength of the relations compared to articles with only a few.

By following the common practice of applying a link weight threshold, we discarded links that were too weak (weight <0.5) and arrived at a network representation of the system under study with only the strong connections left between the authors. The weight threshold was chosen in a way that keeps all the ERC winners in the graph, but still discards as many weak edges as possible. We applied a straightforward method by choosing the minimum among each winner node's largest edge weights, which guarantees that all nodes considered are left in the graph with at least 1 link. The threshold turned out to be a reasonable 0.5, equivalent to the two researchers having at least one article together with at most one other author, or two articles with at most three other authors, or three articles with at most five other authors, etc.

In terms of the general network properties, we examined the link density (the ratio between the number of existing links and the number of possible links), the average shortest path length (over all possible node pairs that are reachable from one to the other), the diameter of the largest connected component (given by the maximum of the shortest path lengths), and the average clustering coefficient (where the clustering coefficient of a single node corresponds to the ratio between the number of existing links between its neighbours and the number of possible links between its neighbours). In addition, we also defined the collaboration distance between a pair of authors as the minimum aggregated inverse weights of the links on the possible paths connecting the two nodes. The intuition behind this definition is that authors who are connected by strong links are likely to either maintain an intensive collaboration or at least tend to work on related subjects, whereas the pairs that can be reached from one to the other only via a series of weak links (resulting in a high distance) are expected to work independently from each other.

## 4 Results

**Fig. 1:**
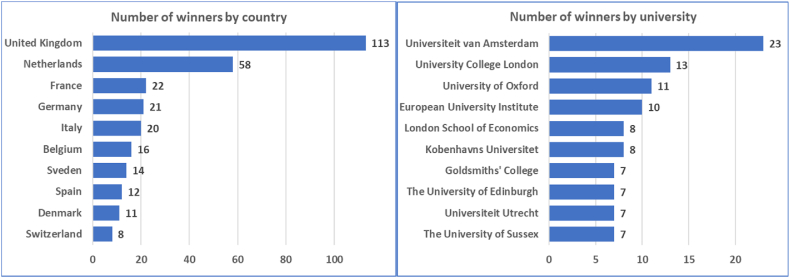
The top ten countries and universities with the most ERC grant winners. We display the raw number of winners in both cases.

Counting ERC grant winners by their nationality shows a clear dominance of Western European countries, especially the United Kingdom and the Netherlands, as indicated in Fig. 1. In the case of the latter, this is even more pronounced when considering its smaller population, and the fact that the leading university is also Dutch (Universiteit van Amsterdam, with almost more ERC winners than the 2nd and 3rd combined). Based on these observations, an interesting question is whether these results were achieved by small, national, grant-focused research groups/institutions, or by certain prominent mentors, or by just overall excellence.

**Fig. 2:**
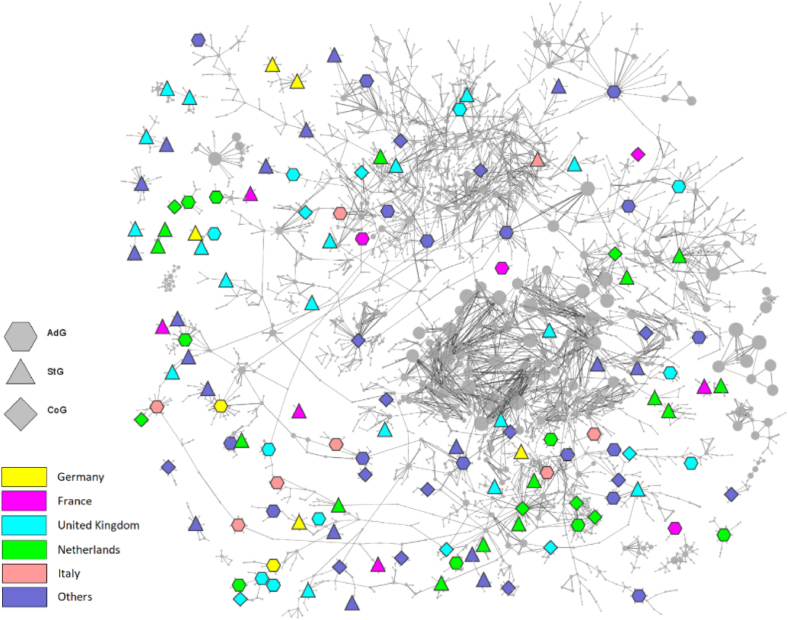
The co-authorship network around the ERC grant winners.

In order to provide an overview, in Fig. 2 we show a layout of the co-authorship network around the ERC winners. The total number of nodes in the examined system was 9950, connected by 23959 links altogether (after applying the link weight threshold described in Section 3). General properties of this network and the most prominent actors (by common centrality measures) are included in the Supplementary material.

Returning to the question of how the high number of English and Dutch winners came to be, defining a “collaboration distance” as the inverse of the collaboration strength (see Section 3) proves useful. With such a distance measure, which is smaller if the authors published more papers together, one can look at the closest ERC winners and see whether they come from the same nation or not. The results for the five leading countries are summarised in Table 1. While the English collaborations can be explained simply by the high number of English grants, the same cannot be said for the Dutch and Italian winners. There seems to be a national bias in these countries, especially in the case of the Netherlands, as there are far more winners from the same nation in the closest five compared to what could be expected from a uniform distribution. This might suggest conscious preparation by the Dutch, by forming strong collaboration groups focusing on research areas important at a European level, eligible to win further grants (a strongly collaborating research community mainly composed of Dutch grantees is shown in Fig. 4).

**Table 1:** The empirical and expected average number of winners with the same nationality among the closest five winners. The expected numbers and the 99.9 % confidence intervals were calculated assuming a uniform distribution of winners in the closest five (i.e., a binomial distribution with n = 5 and p as the ratio of winners from a given nationality). The confidence intervals were estimated using the Wilson method.

	United Kingdom	Netherlands	France	Germany	Italy
Average (empirical)	1.51	1.79	0.18	0.29	0.70
Expected [99.9 % conf.]	1.68 [1.29; 2.13]	0.86 [0.58; 1.25]	0.33 [0.17; 0.62]	0.31 [0.16; 0.61]	0.30 [0.15; 0.59]

The winners correspond to the coloured nodes (where the nationality is coded by the different colours), while the shape of the nodes indicates the different grant types. The size of the nodes (apart from the ERC winners) is controlled by their strength (corresponding to the aggregated link weights connected to the node).

The seven researchers found in the community with the most Dutch grantees are active in applied social sciences concerning communication and information technology, with the leading topics related to the “*Elections, European Parliament, Referendum*”, “*Political Participation, Social Media, Media Use*”, and “*Radical Right, Populist, Right-wing Populism*” according to the SciVal database.

**Fig. 3:**
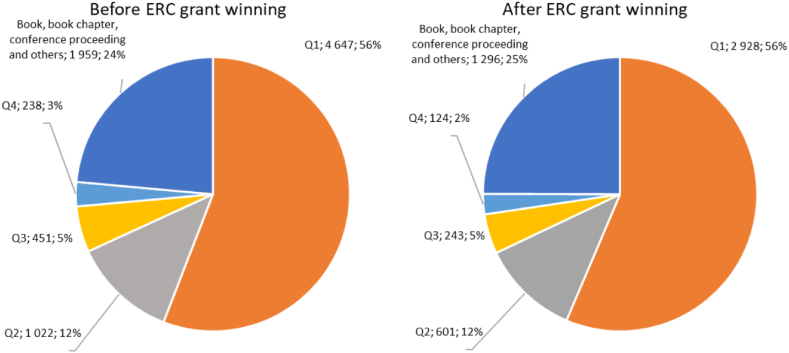
Share of publication types by quality of journal before and after ERC grant winning.

Looking at the quality of the publications of ERC winners, it is no surprise that most of their work is found among the top indexed (Q1) papers (Fig. 3). This is also true before the award and barely changes after it. By delving deeper into the data, a high share of Q1-indexed publications can be observed (71.4 % of the scholarly output between 2011 and 2020) based on the SciVal database, while 45.6 % of the publications were published in the top 10 % journals according to CiteScore. Regarding the impact, 31.6 % of these publications are among the top 10 % of publications worldwide with the highest number of citations between 2011 and 2020. The same data for the EU-27 average in social sciences are 40.5 %, 20.4 %, and 12.9 %, respectively for the same period. The share of the publication in the top 1 % journals is 9 % for the SH2 grantees and 2.7 % for the EU-27 average in social sciences in 2020. This leads to the conclusion that ERC grants are not for “starters” trying to reach the most prestigious scientific platforms, but rather for providing already excelling researchers with the opportunity to pursue their ideas in Europe.

**Fig. 4:**
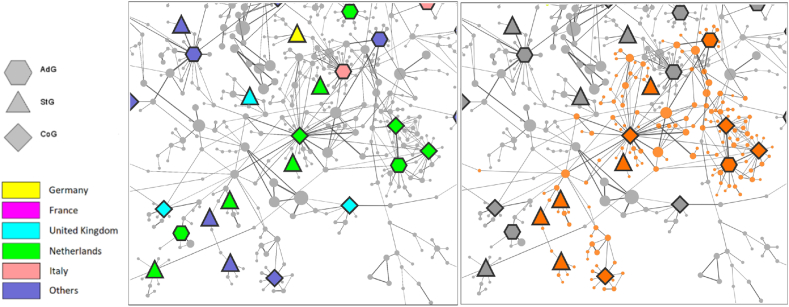
The research community with the most Dutch grant winners.

No significant change is observed after the winning of the grant, meaning that the grant is not an entry into the top-ranked publication platforms, but rather a next step for excelling researchers. The large proportion of books and others is characteristic of social sciences.

Left: Part of the co-authorship network around ERC grantees, where colours and shapes code the nationality and type of grant. Right: Community members highlighted with orange, where ”*community*” refers to a network cluster found by the Louvain community finding algorithm. Both the large number of winners and the high proportion of Dutch (7 out of 11) are unusual for a community of this size.

It is worth noting the extreme width of the distribution in the case of Advanced Grants, which shows consistency with Barabási's 5th law in the sense that success can come at any time. Starting Grants, on the other hand, display a much narrower distribution, and thus have a meaningful average, making an application in this category much more plannable. These distributions were obtained using Kernel Density Estimation.

**Fig. 5:**
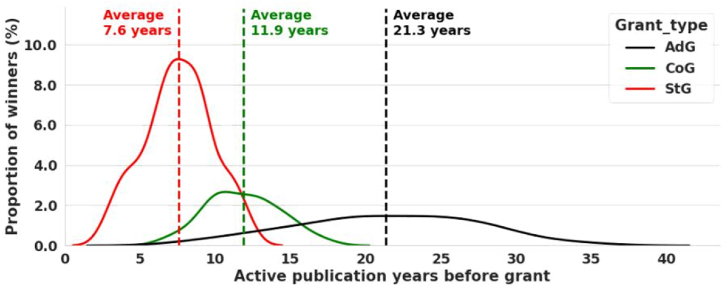
The density distribution of the elapsed time (measured in years) between the first publication and the winning of the grant among the grantees, shown separately for the three grant types.

Although the success of an ERC grant application should mainly depend on the quality of the research proposal itself, there are some clearly visible patterns in the grantees' publications. These draw a career track of successful applicants by quantitative measures, which can serve as guidelines for ambitious social scientists, so that they can have a levelled playing field when applying for a grant. In Fig. 5, we show the distribution of active publication years before winning a grant. It is important to note that the years are calculated from the first publication available in Scopus. Starting Grant winners have the narrowest distribution, with an average of 7.6 years of publication. As applicants must not have their PhD for more than 7 years, this suggests that most of them start publishing at the beginning of the doctoral school, or even during their master's course. Consolidator grants are awarded after 11.9 years of publication on average, while for Advanced Grants, the mean of the active publication years is 21.3. However, in the latter case, the average is not as meaningful because the distribution is extremely wide compared to the previous two cases. Nonetheless, the observed distribution for Advanced Grants is consistent with Barabási's 5th law, which states that with persistence, success can come at any time.

The number of publications of successful applicants can also be quantified. The path of progression can be drawn by plotting the cumulative number.

**Fig. 6:**
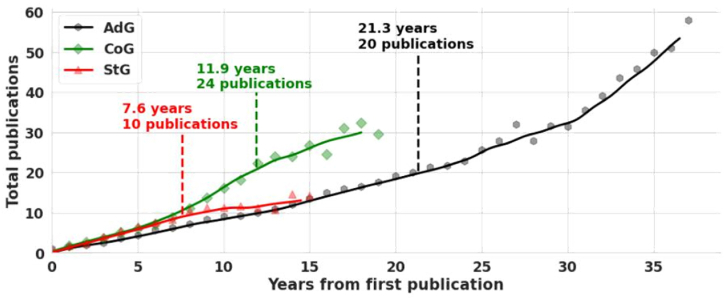
The number of papers published by the winners as a function of their active publication years.

The symbols indicate the average value, with trend lines calculated as moving averages. The vertical dashed lines mark the average winning years for the different grant types. A clear progression path is visible for all grant types, with each starting similarly. The Consolidator Grant seems to be the most competitive out of the three: these researchers already started their careers before the introduction of the grant and now are trying hard to meet the requirements at a pace of publications as a function of the active publication years, as shown in Fig. 6. All three grant applicants start similarly, reaching an average of 10 publications after seven and a half years. For Starting Grants this could already be enough. In the case of Consolidator Grants, there comes a steep increase in the number of publications after the 7.5-year mark. This can be explained by assuming that these researchers started to build their careers before the introduction of the grant, and now they are trying to meet the new standards at an increased pace. Advanced Grant winners also follow a steady trend; however, their publication output falls behind even Starting Grant applicants. This is no surprise, since Advanced Grants are awarded to researchers after significant research achievements, thus have an even looser dependence on the number of publications alone. As Consolidator Grants are very competitive with a high number of publications, and Advanced Grants require an already well-established career, Starting Grants seem to be the most straightforward for an aspiring young social scientist.

**Fig. 7:**
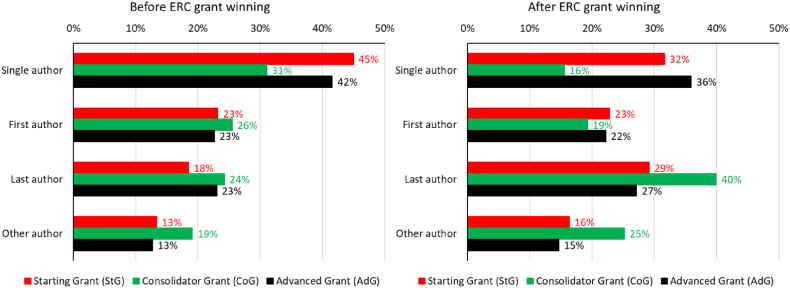
Publication activity before and after the grant from the point of authorship roles.

Besides the publication activity, one can look at the authorship roles of winning applicants as well. The first and single authorship dominates in all categories before winning the grant, as shown in Fig. 7, indicating the significant contribution to the published works by the applicants. In contrast, after winning the ERC grant, there is a significant decrease in the number.

Before winning, single and first authorship dominate in all categories. After obtaining the grant, the winners start to appear as last or other authors more often, becoming more of a support engine driving research communities of single-authored publications (32 %; 16 %; 36 %). This also comes with a notable increase in the proportion of last author publications (29 %; 40 %; 27 %), while other author publications also show a slight increase (16 %; 25 %; 15 %). All these changes (especially the increase in other authorships) signal that the grant has a strong community-shaping power, helping researchers establish or extend their collaboration networks.

**Fig. 8:**
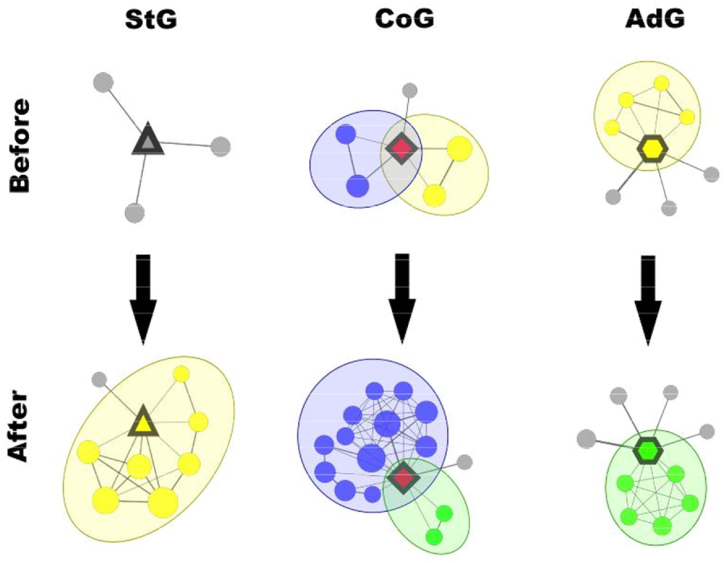
Typical examples of the changes occurring in the ego networks of the winners after receiving the grant.

These shifts in collaboration patterns around a winner can be further explored by looking at their “ego networks” (corresponding to their one-step neighbourhood in the co-authorship network) before and after winning the grant, as illustrated in Fig. 8. In this analysis, we focused on the closely collaborating groups (also called communities, modules, or clusters) in the ego networks. Since an active researcher may work on multiple subjects at the same time, we can expect that some of the winners may also belong to multiple groups simultaneously. Therefore, to allow overlaps between the collaboration groups in a natural way, we extracted these groups using the k-clique percolation algorithm [38], corresponding to a widely known overlapping community finding approach. According to our analysis, the evolution of the intense collaboration groups in the close vicinity of the winners reveals some interesting differences between the three grant types. The statistics related to the detected collaboration groups before and after the grant winning are summarised in Table 2.

**Table 2:** Basic statistics of the ego networks and the collaboration groups found before and after winning the grant. We display the median value for each attribute.

	StG		CoG		AdG	
	Before	After	Before	After	Before	After
#Coauthors	2.5	7	7.5	15	4	7.5
#Groups	0	1	2	2	1	1
Group size	0	3	3	5	3	3

Based on that, for Starting Grants the median number of collaboration groups rises from 0 to 1, and the number of co-authors more than doubles.

The structure of the intensively collaborating groups (indicated by the colours) in the ego networks may undergo significant modifications. Here, the examples show the formation of the first research group (that is typical for StG), expansion of the collaboration groups (that occurs often for CoG), and switching to another research team (that we can observe frequently for AdG) (from 2 to 7).

This reflects the fact that these grants allow relatively young scientists to start a serious endeavour and provide the necessary publicity so that they can find or establish the group they want to work with.

In the case of Consolidator Grants, the number of collaboration groups does not change on average, however, the number of co-authors doubles, and the size of the cooperating groups also increases significantly. Additionally, in 75 % of the cases there were less than 15 % of co-authors who remained in the network after the grant (and in almost half of the cases none remained). These facts prove the success of the grant in forming actively collaborating scientific communities directed towards new research ideas.

Finally, in the case of the Advanced Grants, the collaboration group statistics shown in Table 2 are identical before and after receiving the grant. This is in line with the statistics presented on the share of types of authorship in Fig. 7, as these researchers have already established their careers. However, the number of co-authors is rising considerably, and similarly to Consolidator Grants, in 75 % of the cases less than 20 % of the collaborators were kept after winning. This suggests that the grant allows well-established scientists to conveniently start working on “*groundbreaking, high-risk*” ideas, not necessarily fully in line with previous research.

## 5 Discussion

Three research questions were formulated based on the literature review. The main elements for stating the problem were the concept of academic capital, the Matthew effect, and Professor Barabási's five laws about success. In this section, based on the literature, a discussion of the results is provided.

The first result is the list of institutions and countries that have leading positions regarding the number of ERC grants won. The list of leading countries proves that the United Kingdom, the Netherlands, France, Germany, and Italy have the highest number of grants, all of them being Western European countries. The results demonstrate a significant difference between the Western European and Central Eastern European region, as the latter is represented by only a few countries, including Hungary with 2 grants, the Czech Republic, and Poland with both 1-1 grant. These results are in alignment with Merton's Matthew effect, and university rankings are generally dominated by the well-developed countries of the Western hemisphere. Scanning through the list of leading institutions led by the English and Dutch universities also reflects on the concept of academic capital. As winning an ERC grant comes with a huge responsibility, it should only be given to trustworthy researchers coming from high-quality institutions, capable of reliably providing the necessary tools, technologies, and infrastructure for the success of the project. Note that this could lead to a self-generating phenomenon, since the number of ERC grants won also contributes to the reputation of a university. Thus, it is worth considering how these institutions might have an influential power on the grant scheme. Interestingly, the winners from the aforementioned leading countries seemed to be distributed in a homogeneous manner in the co-authorship network. There were only few cases where winners from the same nationality were observed to be closer in the network than expected (when compared to a uniform distribution; see Table 2). Of these, the Dutch present an extreme case where one might suspect conscious preparation in forming strong national collaboration groups, capable of achieving the grant requirements.

The second result is the mapping of an average ERC winner's career path using quantitative measures. A high (>50 %) portion of top-ranked Q1 articles seems to be common for all grant types. This is consistent with Barabási's 3rd law: future success is determined by previous success multiplied by the fitness of the idea; thus, it is natural for peers to support already excelling researchers in hope of future achievements. The averages for successful grant application were found to be 10 publications and 7.6 active publication years for StG grant winners, 24 publications and 11.9 active years for CoG grant winners, and 21.3 publications and 20 active years for AdG grant winners. In addition to the quality of publications, these can serve as guidelines for researchers thriving to apply for Starting- and Consolidator Grants in social sciences. As Advanced Grants are awarded after wide distribution of active publication years, the above-presented numbers are less characteristic of an average winner. However, the result aligns well with Barabási's 5th law, that with persistence success can come at any time.

The third result is about the change in authorship roles and the surrounding co-authorship network before and after winning the ERC grant. Before the grant, we can see a high share of single-authored (45 %; 31 %; 42 %) and first-authored (23 %; 26 %; 23 %) publications in every grant type, while after the successful ERC application, the share of last authored (29 %; 40 %; 27 %) and other authored (16 %; 25 %; 15 %) articles increases significantly. The shift to last and other authorship indicates that an extended collaboration system forms around the winners, thus demonstrating the strong community shaping power of the ERC grant scheme. This is also confirmed by analysing the changes in the co-authorship network, as the formation of new groups and intensifying intra-group collaboration patterns can be observed in the case of all three grant types. It could also be determined from network analysis that after winning it is not unusual to start working with completely new research groups, allowing to enter different research fields even for well-established scientists.

To sum it up, we carried out a study focusing mainly on the research output of the ERC grant winners, following a methodology based on scientometric tools and network science. We were able to identify characteristic patterns of the career paths leading to European recognition of excellence by examining quantitative metrics in publication output. Moreover, we showed how the award promotes collaboration around the winner, making the support of a promising researcher favourable for everyone. The obtained results are also consistent with the laws of success by Barabási and the Mathew effect by Merton. Further related interesting fields that could be explored in future works, are the study of the distribution of winners among research topics, the distribution of research topics among collaboration groups, or the collaboration patterns between the top-ranked institutions.

## Declarations

The authors have no conflicts of interest to declare that are relevant to the content of this article.

## Data availability statement

Data will be made available on request.

## CRediT authorship contribution statement

**Anna Urbanovics:** Writing – review & editing, Writing – original draft, Supervision, Investigation, Formal analysis, Conceptualization. **István Márkusz:** Writing – original draft, Software, Methodology, Data curation. **Gergely Palla:** Visualization, Supervision, Project administration, Methodology. **Péter Pollner:** Supervision, Project administration, Methodology, Conceptualization. **Péter Sasvári:** Writing – review & editing, Supervision, Project administration, Methodology, Investigation, Data curation.

## Declaration of competing interest

The authors declare that they have no known competing financial interests or personal relationships that could have appeared to influence the work reported in this paper.
