# Supplementary material for: Path of excellence: A co-authorship network analysis of European Research Council grant winners in social sciences
Source: Heliyon. 2024 Jun 19;10(12):e32403. doi: 10.1016/j.heliyon.2024.e32403 (PMC11252591; doi:10.1016/j.heliyon.2024.e32403)
Supplement: Multimedia component 1 [file mmc1.docx]

# Supplementary materials

Supplementary table 1: **General properties of co-authorship network**. The relatively high average clustering coefficient and the low overall link density indicate that this is a globally sparse, locally dense network, similarly to social networks in general. (The definitions of the shown quantities are given in Section 3)

| #Nodes | #Edges | Link density | Avg. shortest path length | Diameter | Avg. cluster coefficient |
| --- | --- | --- | --- | --- | --- |
| 9950 | 23959 | 4.84e-4 | 7.47 | 22 | 0.40 |

Supplementary table 2: **Most prominent actors by common centrality measures**. The last column in each table contains the ERC panel won (if any). The two centralities were calculated using the inverse of the collaboration strength as distance (see Section 3). As expected, the number of ERC winners is relatively high among the most central actors (in case of SH2 it is 1.4 and 5.7 times more than expected from a uniform distribution for closeness and betweenness, respectively)

| Author | Closeness | ERC panel |  | Author | Betweenness | ERC panel |
| --- | --- | --- | --- | --- | --- | --- |
| Mackenbach J.J.P. | 0.2826 |  |  | Mackenbach J.J.P. | 0.2293 |  |
| Van Lenthe F.J. | 0.2818 |  |  | Van Lenthe F.J. | 0.1630 |  |
| Kunst A.E. | 0.2811 |  |  | Kamphuis C.B.M. | 0.1563 |  |
| Kamphuis C.B.M. | 0.2802 |  |  | Nijkamp P.J. | 0.1536 |  |
| Avendano Pabon M. | 0.2780 | SH2 (2010) |  | Van Den Bergha J.C.J.M. | 0.1490 | SH2 (2016), SH7 (2022) |
| Ettema D.F. | 0.2776 |  |  | Ettema D.F. | 0.1481 |  |
| Kawachi I. | 0.2774 |  |  | Rietveld P. | 0.1297 |  |
| Timmermans H.J.P.H. | 0.2766 | SH3 (2008) |  | Timmermans H.J.P.H. | 0.1294 | SH3 (2008) |
| P Subramanian S.V. | 0.2763 |  |  | Van Wee G.P.W. | 0.1225 |  |
| Nusselder W.J. | 0.2759 |  |  | Kawachi I. | 0.1203 |  |
| Hofman A. | 0.2759 |  |  | Molin E.J.E. | 0.1145 |  |
| Stronks K. | 0.2759 |  |  | De Vreese C.H. | 0.1118 | SH2 (2014) |
| Arentze T.A. | 0.2758 |  |  | Kunst A.E. | 0.1009 |  |
| Molin E.J.E. | 0.2757 |  |  | Neijens P.C. | 0.0922 |  |
| Jaddoe V.W.V.W. | 0.2755 | LS7 (2014) |  | Kivimaki M.S.M. | 0.0915 |  |
| Van Wee G.P.W. | 0.2752 |  |  | Van Den Putte B.V. | 0.0911 |  |
| Rietveld P. | 0.2751 |  |  | Marmot M.G. | 0.0844 | LS7 (2010) |
| Nijkamp P.J. | 0.2750 |  |  | Jordan A.J. | 0.0812 | SH2 (2019) |
| Marmot M.G. | 0.2749 | LS7 (2010) |  | Avendano Pabon M. | 0.0774 | SH2 (2010) |
| Shipley M.J. | 0.2744 |  |  | Savenije H.H.G. | 0.0763 |  |
